# Iron Deficiency-Induced Hair Loss Is Associated with ROS-Mediated Disruption of Wnt/β-Catenin Signaling

**DOI:** 10.3390/nu18142321

**Published:** 2026-07-15

**Authors:** Sang-Ah Kwon, So Young Bu, Yeon-Hee Kim, Joo Weon Lim, Christopher D. Vulpe, Seung-Min Lee

**Affiliations:** 1 Department of Food and Nutrition, Yonsei University, Seoul 03722, Republic of Korea; 2 Department of Food and Nutrition, Daegu University, Gyeongsan-si 38453, Republic of Korea; 3 Division of Cancer Epidemiology and Prevention, National Cancer Center, Goyang-si 10408, Republic of Korea; 4 Department of Physiological Sciences, Center for Environmental and Human Toxicology, University of Florida, Gainesville, FL 32611, USA; 5 Research Institute of Science for Aging, Yonsei University, Seoul 03722, Republic of Korea

**Keywords:** iron deficiency, hair loss, reactive oxygen species, oxidative stress, Wnt/β-catenin

## Abstract

Background: Gestational iron requirements may lead to maternal iron deficiency, increasing susceptibility in offspring. In mice, maternal iron deficiency induces hair loss in pups, but mechanisms remain poorly characterized. Objective: This study investigates mechanisms underlying maternal iron deficiency-induced abnormal hair growth and the impact of iron supplementation on pups’ hair development. Methods: Pregnant C57BL/6J mice (nine-week-old, second week of gestation) were randomly assigned to a standard AIN-76 diet (control, CTRL) or an iron-deficient AIN diet (ID) until parturition and weaning. Offspring were maintained on the same diet as their mothers. At six weeks, hair loss was examined in one set (CTRL1 and ID), while the remaining were transitioned to an iron-replete AIN-76 diet (CTRL2 and IDN) for two additional weeks. An in vitro study with human follicle dermal papilla cells (HFDPC) via deferoxamine (DFO) treatment was performed. Results: ID offspring exhibited truncal hairlessness, reduced body size, and abnormal follicular morphology compared to CTRL1, while IDN demonstrated hair regrowth comparable to CTRL2. ID skin tissues had reduced Wnt/β-catenin signaling, elevated oxidative stress markers, and activation of caspase-3, nuclear factor kappa B (NF-κB), and transforming growth factor-beta (TGF-β) signaling, all reversed by iron supplementation. DFO-treated HFDPCs demonstrated increased cellular and mitochondrial reactive oxygen species (ROS), diminished Wnt/β-catenin signaling, activation of caspase-3, NF-κB, and TGF-β signaling pathways. *N*-acetylcysteine pretreatment abrogated DFO-induced alterations in Wnt/β-catenin signaling and apoptosis, suggesting ROS mediates iron deficiency-induced hair loss. Conclusions: Early iron deficiency may have impaired hair growth through increased ROS production, reduced Wnt/β-catenin signaling, and enhanced apoptotic signals, while postnatal iron supplementation could reverse these abnormalities.

## 1. Introduction

Iron deficiency is the most common form of malnutrition throughout the lifespan [1]. Globally, 38% of pregnant women are affected by iron deficiency [1]. Iron requirement increases during pregnancy. Approximately, 40.1% of pregnant women were anemic worldwide [2]. The Institute of Medicine recommends an additional iron intake of 27 mg/day during pregnancy to meet the increased iron demand [3]. Maternal iron deficiency results in low iron status in the fetus and the neonate, increasing the risk of anemia in infants and young children [4]. In addition to anemia risk, early exposure to iron deficiency may lead to neurological disorders and cognitive deficits, which are difficult to reverse in adulthood [5]. Iron deficiency has been observed in clinical studies as a common cause of hair loss in children [6]. Women of childbearing age experiencing iron deficiency have reported to develop diffuse hair loss [7]. Low serum ferritin levels, which reflect depleted iron stores, have been observed in patients with chronic telogen effluvium (TE)—characterized by hair thinning and shedding—and alopecia areata (AA), an autoimmune disorder causing patchy hair loss [8].

Studies in genetically modified mouse models further highlight the importance of iron in hair health. Transgenic mice overexpressing liver hepcidin exhibit severe iron deficiency anemia and complete truncal hair loss [9]. Similarly, mice with a genetic deletion of ferroportin-1 in macrophages displayed hair loss and dilation of hair follicles at birth, which coincided with iron deficiency [10]. Moreover, a characteristic hair loss phenotype was exhibited in pups born to hephaestin knockout (or *sla*) dams, attributed to iron-deficient milk resulting from impaired maternal iron metabolism [11,12]. This phenotype further supports the relationship between iron transport and hair follicle biology [13]. In humans, significant hair regrowth has been observed in patients with hair loss following iron supplementation therapy [14]. Taken together, these findings suggest that iron sufficiency is crucial for normal hair development and growth. However, the specific biological pathways through which iron deficiency leads to hair loss have not fully been investigated yet. Especially, the impact of iron status at the local tissue level, such as in the dermal papilla, warrants further exploration under iron-deficient conditions induced by dietary restriction rather than genetic modifications.

Therefore, we assessed whether early-onset iron deficiency during gestation, due to an iron-insufficient maternal diet, could induce hair loss. We then investigated the mechanisms by which iron deficiency causes hair loss and iron supplementation reverse the effects. Additionally, we performed an in vitro study to investigate the mechanisms underlying iron deficiency-induced hair loss.

## 2. Materials and Methods

### 2.1. Animals and Diet

**Figure 1:**
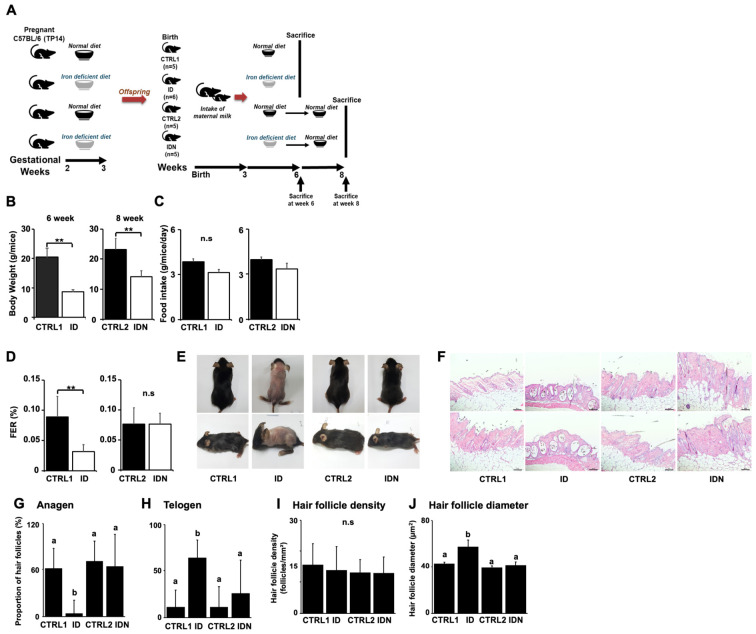
Early exposure to an iron-deficient diet led to weight and hair loss, but iron supplementation restored hair growth in pup mice. C57BL/6J pups born to iron-deficient mothers were fed either an iron-sufficient (CTRL1) or iron-deficient diet (ID) for six weeks and then fed a normal iron-sufficient diet for two more weeks (CTRL2 and IDN). (**A**) The timeline of the study (**B**) body weight at sacrifice (**C**) average food intake, (**D**) and food efficiency ratio (FER) of CTRL and ID groups, CTRL2 and IDN groups during the study period. Hair growth- and follicle formation-preventing effect of dietary iron deficiency in mice offspring. The dorsal skin of C57BL/6J mice were fed with either iron sufficient (CTRL1) or deficient diet (ID) for 6 weeks and then fed with normal iron sufficient diet for 2 more weeks (CTRL2, IDN). (**E**) The dorsal skin was photographed at sacrifice (6th and 8th weeks). (**F**) Skin tissues of mice in each group were stained using H&E staining. (**G**) Proportion of hair follicles in the anagen stage. (**H**) Proportion of hair follicles in the telogen stage. (**I**) Hair follicle density (follicles/mm^2^). (**J**) Hair follicle diameter (μm). Data in panels (**B**–**I**) are presented as mean ± SD, whereas data in panel (**J**) are presented as mean ± SE. Data in panels (**B**–**D**) were analyzed using Student’s *t*-test. Data in panels (**G**–**J**) were analyzed using one-way analysis of variance (ANOVA) followed by Tukey’s multiple comparisons test. ** *p* < 0.01; n.s., not statistically significant. FER = (Weight gain/food intake) × 100. Different lowercase letters indicate statistically significant differences among groups (*p* < 0.05, one-way ANOVA followed by Tukey’s multiple comparisons test).

Four female C57BL/6J mice (nine weeks old) in their second week of pregnancy were purchased from DooYeol Biotech (Seoul, Republic of Korea) and housed individually in cages with free access to autoclaved distilled water. The animals were maintained under a 12 h light/dark cycle in a temperature- and humidity-controlled pathogen-free rodent colony room. Mice were divided into two groups: normal AIN-76A diet (35 mg/kg wet iron, FeSO_4_·7H_2_O) and AIN-76A-based iron-deficient diet (3 mg/kg wet iron, FeSO_4_·7H_2_O). The diet composition is listed in Supplementary Table S1, and the study scheme is shown in Figure 1A. For a total of two weeks during gestation, the pregnant mice were fed an iron-deficient diet for one week (which corresponds to 27–40 weeks (3rd trimester) of gestation in humans) which continued after giving birth and until weaning. The pups were suckled by the dam while on an iron-deficient diet, and after weaning newborn mice were maintained on an iron-deficient diet until the ages of six weeks (ID group). The mice in the ID group at 6 weeks of age were sacrificed for analysis, and the remaining mice were switched to a normal diet for another two weeks (the IDN group). Control animals corresponding to either the ID group (CTRL1) or the IDN group (CTRL2) were fed the iron-sufficient AIN-76A diet throughout the respective six-week and eight-week study periods. The offspring were sacrificed at weeks 6 (CTRL1, *n* = 5; ID, *n* = 6) and 8 (CTRL2, *n* = 5; IDN, *n* = 5). At sacrifice, skin (approximately 3 cm^2^ was collected including all dermal layers) and liver tissues were collected and frozen immediately in liquid nitrogen and stored at −80 °C until analysis. All animal experimental protocols were reviewed and approved by the Institutional Animal Care and Use Committee of the Yonsei Laboratory Animal Research Center (IACUC-A-201804-723-01). All experiments were performed in accordance with the Korean Food and Drug Administration guidelines.

### 2.2. Histological Examination of Hair Follicles

Central dorsal skin parallel to the vertebral line was cut in a rectangular form, fixed in 10% formalin, sliced in 4-μm-thick longitudinal sections, and stained with hematoxylin and eosin. Stained sections obtained from all animals were examined under a Nikon Eclipse Ti microscope (Nikon, Kobe, Japan) at 40× magnification. Images of hair follicle morphology were captured using a digital camera (Nikon Digital Sight DS-U3; Kobe, Japan) and analyzed using a commercially available imaging program (TOMORO Scope Eye; Seoul, Republic of Korea).

Hair follicle density was quantified using ImageJ software (version 1.54g; National Institutes of Health, Bethesda, MD, USA). The analyzed tissue area was measured using ImageJ, and only longitudinally sectioned hair follicles were manually counted, whereas transversely sectioned follicles were excluded. Hair follicle density was calculated as the number of hair follicles per unit area (follicles/mm^2^). Hair follicle diameter was measured at the widest point of each longitudinally sectioned hair follicle, and the mean diameter for each mouse was used for statistical analysis. Multiple non-overlapping fields were analyzed for each mouse, and the mean value was used for statistical analysis.

Hair follicle cycling was evaluated histomorphometrically according to the classification system described by Müller-Röver et al. [15]. Longitudinally sectioned hair follicles were classified as anagen, catagen, or telogen based on their characteristic histological features. The proportion of follicles in each hair cycle stage was expressed as the percentage of the total number of hair follicles counted. For each experimental group, skin samples from four mice were analyzed, and at least four non-overlapping microscopic fields per mouse were evaluated.

### 2.3. Ferrozine Assay

Liver tissues (50 mg) were homogenized using mortal, pestle, and a 22 G syringe in the lysis buffer containing 50 mM Tris-HCl buffer, pH 7.4, 150 mM sodium chloride, 1 mM ethylenediaminetetraacetic acid (EDTA), 1% Triton X-100, 1 mM phenylmethanesulfonyl fluoride (PMSF), 1 mM benzamidine, 1 mg/mL leupeptin, 10 mM chymostatin, 1 mg/mL antipain, and 1 mg/mL pepstatin A. Homogenized tissue was centrifuged at 10,000× *g* for 5 min at 4 °C. Sample aliquots (180 µL) were mixed with the same volume of 10 mM HCl (180 µL) and iron-releasing agent (180 µL) composed of 1.4 M HCl and 4.5% (*w*/*v*) KMnO_4_. The mixture was incubated at 60 °C for 2 h. Then, the mixture was brought to room temperature and incubated for 30 min in an iron-detection reagent (60 µL) consisting of 6.5 mM ferrozine (Sigma-Aldrich, St. Louis, MO, USA), 6.5 mM neocuproine (Sigma-Aldrich, St. Louis, MO, USA), 2.5 mM ammonium acetate (Sigma-Aldrich, St. Louis, MO, USA), and 1 M ascorbic acid (Sigma-Aldrich, St. Louis, MO, USA). The absorbance was measured at 550 nm using a 96-well plate reader (Infinite M200 Pro; Tecan Group Ltd., Männedorf, Switzerland).

### 2.4. Reverse Transcriptase and Quantitative Polymerase Chain Reaction (qPCR)

Total RNA was extracted from the skin and liver tissues through homogenization using TRIzol reagent (MRC, Cincinnati, OH, USA). Extracted RNA was reverse transcribed using ImProm II reverse transcriptase (Promega, Madison, WI, USA), and cDNA was synthesized using BioFact™ RT-Kit (BIOFACT, Daejeon, Republic of Korea) according to the manufacturer’s protocol. Quantitative PCR was performed in the Bio-Rad CFX Real-time system (Bio-Rad, Hercules, CA, USA) using 5× HOT FIREPOL^®^ EvaGreen^®^ qPCR Supermix (Solis biodyne, Tartu, Estonia) according to the manufacturer’s cycling conditions. A relative quantification platform using the comparative 2−(ΔΔCq) method was used to quantify the gene of interest relative to β-actin mRNA expression. All primers used are listed in Supplementary Table S2.

### 2.5. Protein Extraction and Western Blotting

Liver and skin tissues (30 mg) or a cell culture sample were homogenized at 4 °C in RIPA buffer with 50 mM sodium pyrophosphate, 50 mM sodium fluoride, 1 mM PMSF, 2 μg/mL aprotinin, and 1 μg/mL leupeptin. Whole-tissue homogenates were centrifuged at 13,000× *g* for 20 min at 4 °C. The nuclear protein fraction of skin tissues (30 mg) or cell culture samples was obtained using an ice-cold fractionation buffer (500 μL) containing 1 M HEPES (pH 7.9), 1 M MgCl_2_, 1 M KCl, 1 M dithiothreitol (DTT), 0.1 M PMSF, and 10% NP-40. After 15 min of incubation on ice, the supernatant was collected through centrifugation at 13,000× *g* for 10 min at 4 °C. Then, 100 μL of a high-sodium buffer containing 1 M HEPES (pH 7.9), 50% glycerol, 1 M NaCl, 1 M MgCl_2_, 0.5 M EDTA, 0.1 M PMSF, and 1.0 M DTT was added, vortexed, and incubated on ice for 30 min. Then, 100 μL of a hypotonic buffer containing 20 mM HEPES (pH 7.9, 1 M), 20% glycerol, 50 mM KCl, 0.2 mM EDTA (0.5 M), 0.5 mM PMSF (0.1 M), and 0.5 mM DTT (1 M) was added and centrifuged at 13,000× *g* for 10 min at 4 °C. The supernatant (nuclear fraction) was collected for protein quantification and subjected to Western blotting. Protein concentrations in whole tissue/cell lysates or nuclear fractions were measured using a protein assay dye reagent concentrate (Bio-Rad, Hercules, CA, USA), according to the manufacturer’s instructions. The quantified proteins were separated using 10% sodium dodecyl sulfate-polyacrylamide gel electrophoresis and transferred onto nitrocellulose membranes (Amersham, Buckinghamshire, UK). The membranes were blocked with 5% skim milk in phosphate-buffered saline (PBS), incubated with primary antibodies (diluted 1:1000) overnight at 4 °C, and washed two times in PBS/0.05% Tween 20 and once in PBS at RT. Anti-ferritin heavy chain (Santa Cruz-sc376594, Dallas, TX, USA), anti-TGF-β (R&D systems-mab1835, Minneapolis, MN, USA), anti-smad2 (Santa Cruz-sc101153, Dallas, TX, USA), anti-phospho-smad2 (Cell signaling-3108, Danvers, MA, USA), anti-cleaved caspase-3 (Cell signaling-9661, Dancers, MA, USA), anti-cleaved caspase-9 (Cell signaling-9505, Danvers, MA, USA), anti-TNF-α (Santa Cruz-sc130349, Dallas, TX, USA), anti-β-catenin (Santa Cruz-sc376841, Dallas, TX, USA), anti-NF-κB (Cell signaling-8242, Danvers, MA, USA), anti-phospho-NF-κB (Cell signaling-3033, Danvers, MA, USA), anti-lamin B (Santa Cruz-sc374015, Dallas, TX, USA) and anti-actin (Santa Cruz-sc376421, Dallas, TX, USA) were used as primary antibodies. After washing, the membranes were incubated with either a secondary goat anti-rabbit antibody (EMD Millipore-2884131, Burlington, MA, USA) or a secondary goat anti-mouse antibody (Bio-Rad Laboratories-1706516, Hercules, CA, USA) in PBS/0.05% Tween 20 containing 2.5% skim milk for 2 h. The blots were developed using the chemiluminescent detection reagent WESTSAVE-UpTM (AbFrontier, Seoul, Republic of Korea), according to the manufacturer’s instructions. Protein images were captured using an AE-9300 Ez-Capture system (ATTO, Tokyo, Japan), and protein band density was quantified using ImageJ software (MRC, Cincinnati, OH, USA).

### 2.6. Determination of Mitochondrial DNA (mtDNA) Copy Number

Total DNA was isolated from the skin tissues using the HiGene™ Genomic DNA Prep Kit (Biofact, Daejeon, Republic of Korea), as per the ‘for Animal Tissue’ option, according to the manufacturer’s instructions. The mtDNA copy number was measured using real-time PCR, and specific primer sets were designed to amplify the mitochondrial and nuclear genomes. For mtDNA quantification, the mitochondrial cytochrome c oxidase II (*COXII*) gene was detected using primers with sequences described in Supplementary Table S2. Nuclear DNA was quantified using primers targeting the beta-2-microglobulin (*B2M*) gene. The PCR reaction mixture contained 20 ng of DNA template, 10 pmol of each primer, and 10 μL of SsoAdvanced™ Universal SYBR^®^ Green Supermix (Bio-Rad, Hercules, CA, USA) in a final volume of 20 μL. PCR was performed on a Bio-Rad CFX96 real-time PCR detection system under the following conditions: initial denaturation step at 95 °C for 3 min, followed by 40 cycles of 95 °C for 10 s (denaturation) and 55 °C for 30 s (annealing/extension). According to a previously described method [16], the relative mtDNA copy number was quantified using the difference in threshold cycle number (Ct) between the nuclear DNA gene *B2M* and the mitochondrial *COXII* gene. This difference denoted as ΔCt was calculated using the equation ΔCt = Ct (B2M) − Ct (COXII). The mtDNA copy number was then determined by the formula: Contents of mtDNA = 2 × 2ΔCt.

### 2.7. Analysis of MDA

Liver and skin tissues (200 mg) were homogenized in a 0.15 M KCL solution. The homogenates and 10% trichloroacetic acid were mixed (1:2), and centrifuged at 1500× *g* for 15 min at 4 °C. The supernatant was then resuspended in trichloroacetic acid. After placing in a water bath (95 °C) for 15 min, the samples were centrifuged at 1000× *g* for 10 min at 18 °C. The optical density of the supernatants was measured at 532 nm with an Infinite^®^ 200 PRO, Tecan, Switzerland.

### 2.8. Cell Culture

Human follicle derma papilla cells (HFDPCs) were purchased from CEFO (Seoul, Republic of Korea). We used an HFDPC-specific growth medium (CEFO Co., Ltd.) according to the manufacturer’s instructions. The cells were passaged every 3–4 days and incubated at 37 °C in a 5% CO_2_ incubator. HFDPCs were seeded in a 60 mm culture dish to make 4 × 10^5^ cells/well and incubated in complete Human dermal papilla growth medium (CEFO BIO, Seoul, Republic of Korea) containing 10% fetal bovine serum (Gibco, Grand Island, NY, USA) and 1% streptomycin/penicillin for 24 h. For protein analysis, water (control), 100 μM deferoxamine mesylate (DFO) or 500 μM DFO and 100 μM of *N*-acetylcysteine (NAC) were treated and incubated for 24 h. Then, proteins were extracted as described above and further analyzed.

### 2.9. Cell Viability Measurement

Briefly, HFDPCs (4 × 10^5^) were seeded into 6-well plates and incubated overnight, and then cells were treated with either water (control) or different concentrations of DFO (100 and 500 μM) for 24 h. Cells belonging to the NAC-treated group were treated with 100 μM NAC for 1 h before treatment with 500 μM DFO. After treatment, MTT (3-(4,5-Dimethylthiazol-2-yl)-2,5-diphenyltetrazolium bromide) reagent (1 mg/mL) (Sigma-Aldrich, St. Louis, MO, USA) was added to each well and incubated for an additional 4 h. The medium was then removed, and formazan crystals produced from MTT were solubilized with dimethyl sulfoxide. The absorbance was measured at 570 nm using a microplate reader (Molecular Devices, Sunnyvale, CA, USA).

### 2.10. Immunofluorescence

HFDPCs (1 × 10^5^ cells) were seeded on a glass slide (Lab-Tek chamber slide, Thermo Fisher Scientific (Nunc), Waltham, MA, USA), nested in a 6-well plate, and treated with DFO and/or NAC for 24 h. NF-κB and β-catenin were detected using confocal microscopy. For this purpose, the cells were fixed with 4% formaldehyde, blocked with 1% BSA and 0.1% gelatin for 1 h, and incubated with either NF-κB or β-catenin antibody for 1 h. After washing with PBS, the cells were treated with FITC-conjugated mouse anti-goat IgG antibody (Santa Cruz Biotechnology) for 1 h. For nucleus detection, five µg/mL of 4′,6′-diamidino-2-phenylindole (DAPI) in blocking solution was added before cells were examined by laser scanning confocal microscope (Zeiss LSM510, Carl Zeiss AG Corporate, Oberkochen, Germany).

### 2.11. Assessment of Total Intracellular Reactive Oxygen Species (ROS)

Intracellular superoxide radicals were estimated using the mitoSOX Red mitochondrial superoxide indicator (Invitrogen Corporation, Carlsbad, CA, USA). HFDPCs were seeded at a density of 1 × 10^5^ cells/well in 60 mm plates and incubated overnight. The cells were treated with DFO and/or NAC for 24 h. A freshly prepared 1 mM MitoSOX solution was added to the cells, followed by further incubation for 30 min. The solution mixed medium was removed, and the cells were rinsed with 1 mL of 1× PBS. The cells were then scraped, and their fluorescence intensity was determined (excitation at 514 nm and emission at 585 nm) using a Multilabel Plate Reader (Victor5, PerkinElmer, Waltham, MA, USA). Cellular ROS levels were calculated according to the manufacturer’s instructions, and the values were expressed based on total protein content.

### 2.12. Quantitation of Mitochondrial ROS

HFDPCs were seeded at 1 × 10^5^ cells/well in 60 mm plates and incubated overnight. Cells were then treated with DFO and/or NAC for 24 h and incubated for 30 min with 10 μM 2′,7′-dichlorofluorescin diacetate solution. The medium was then removed and washed with PBS, the cells were detached using a scraper, and fluorescence was measured using a Multilabel Plate Reader (Victor5, PerkinElmer, USA). Mitochondrial ROS levels were calculated according to the manufacturer’s instructions, and the values were normalized to the protein level.

### 2.13. Analysis of Mitochondrial Membrane Potential (MMP)

Cells were seeded and cultured on glass coverslips coated with poly-L-lysine To measure the changes in the MMP. Cells were treated with DFO and/or NAC, and then incubated with the dye reagent in 1:100 (*v*/*v*) (JC-1, Cayman Chemical Company, Ann Arbor, MI, USA) for 20 min. After removing the dye solution, the cell-attached slide was dried for 15 min at RT, and washed twice with PBS. The cells were mounted using a mounting solution (M-7534; Sigma-Aldrich). Red or green fluorescence was examined using a laser scanning confocal microscope (LSM 980, Carl Zeiss Inc., Oberkochen, Germany). JC-1 fluorescent images were analyzed with ImageJ 5.0 software (National Institutes of Health, Bethesda, MD, USA) and expressed as the percentage ratio of fluorescence densities. The average intensity per individual cell was determined, and more than 50 cells per experimental group were examined.

### 2.14. Cell Cycle Analysis

HFDPCs were seeded at 1 × 10^5^ in 6-well plates, cultured overnight, and then treated with 100 or 500 μM DFO for 24 h. After treatment, the cells were washed with PBS, harvested with trypsin–EDTA, and pelleted through centrifugation at 300× *g* for 5 min. The cells were resuspended and fixed with 70% ethanol at −20 °C overnight. The cells were then washed twice with PBS and centrifuged at 500× *g* for 5 min. The pellet was broken up by vortexing and then resuspended in 250 μL of PBS containing propidium iodide (50 μg/mL) and RNase A (0.5 μg/mL) and incubated for a further 30 min in the dark. Finally, the cells were analyzed using flow cytometry (BD FACSCalibur, BD Biosciences, San Jose, CA, USA).

### 2.15. Tunnel (Terminal Deoxynucleotidyl Transferase dUTP Nick End Labeling) Assay

TUNEL staining was used to evaluate cell apoptosis using a TUNEL assay kit (Promega, USA), according to the manufacturer’s instructions. Briefly, the cells were fixed with 4% formaldehyde solution in PBS for 25 min, and the coverslips were washed twice with PBS for 5 min. The cells were permeabilized with 0.2% triton X-100 for 5 min. After washing the cells twice with PBS for 5 min each, the equilibration buffer was applied to the cells for 5 min before incubation, and the solution was removed. The TUNEL reaction mixture was then added to the slides. The slides were incubated in a humidified chamber at 37 °C in the dark for 1 h, and the reaction was stopped by washing three times with PBS in a 2× SSC buffer. After drying, the slides were stained with DAPI in a mounting medium and placed at 4 °C with a cover slip, protected from light prior to microscopy. Finally, fluorescence was measured using a Multilabel Plate Reader (Victor5, PerkinElmer, USA).

### 2.16. Intracellular ATP Detection

ATP levels were determined according to the manufacturer’s instructions (ATP Assay Kit; Invitrogen Corporation, Carlsbad, CA, USA). Briefly, HFDPCs were seeded at 5 × 10^4^, treated with DFO and/or NAC for 24 h, and washed with PBS. Then, 0.5% Triton X-100 in 100 mM/L glycine buffer (pH 7.4) was added to the cells and shaken at 4 °C for 20 min. The supernatant was then transferred to a microtube and centrifuged at 14,000× *g* for 20 min, and the luminescence intensity was measured using a Multilabel Plate Reader (Victor5, PerkinElmer, USA).

### 2.17. Statistical Analysis

Statistical analyses were performed using the SPSS software (version 12.0; SPSS, Chicago, IL, USA). Data is represented as the mean ± standard error (SE) or deviation (SD). The means of the experimental groups were compared using a one-way analysis of variance or Student’s *t*-test. *p*-values < 0.05 were considered statistically significant.

## 3. Results

### 3.1. Effect of Iron Deficiency and Postnatal Supplementation on Body Weight, Hair Growth, and Follicular Morphology

The timeline of the study is illustrated in Figure 1A. The body weight of the ID pups was 57.7% of the CTRL1 pups at six weeks post-birth, and this reduction was not fully restored after two weeks on a normal AIN-76A diet (Figure 1B). Because the average daily food intake did not significantly differ between the ID and IDN groups and their respective controls (Figure 1C), the food efficiency ratio (bodyweight gain per food intake) was significantly lower in the ID group compared to the CTRL1 group (Figure 1D). The ID group exhibited little to no hair coat on the trunk of the body, whereas the CTRL1 group exhibited full hair coverage (Figure 1E). Approximately 9–10 days after transition to an iron-sufficient AIN-76A diet, the IDN group displayed hair growth, in terms of color and length, similar to the CTRL2 group (Figure 1E). The ID group demonstrated fewer and abnormally dilated hair follicles compared to the CTRL1 group, while the IDN group exhibited an increased number of hair follicles compared to the ID group (Figure 1F). Additionally, hair cycle analysis revealed that the ID group exhibited significant reduction in the proportion of anagen hair follicles, accompanied by an increase in telogen follicles compared to other groups which did not exhibit the hair loss phenotype (Figure 1G,H). Notably, hair follicles in the catagen phase were rarely detected or remained below the threshold of reliable quantification across all groups. Meanwhile, total hair follicle density did not differ significantly among the experimental groups (Figure 1I), while the hair follicle diameter was significantly greater in the ID group than in the CTRL1 and CTRL2 groups (Figure 1J). These findings indicate that early iron deficiency impairs normal body weight gain and hair growth, and that two weeks of postnatal iron supplementation was sufficient to restore hair growth.

### 3.2. Effect of Iron Deficiency and Postnatal Supplementation on Tissue Iron Levels and Expression of Iron Regulatory Genes

**Figure 2:**
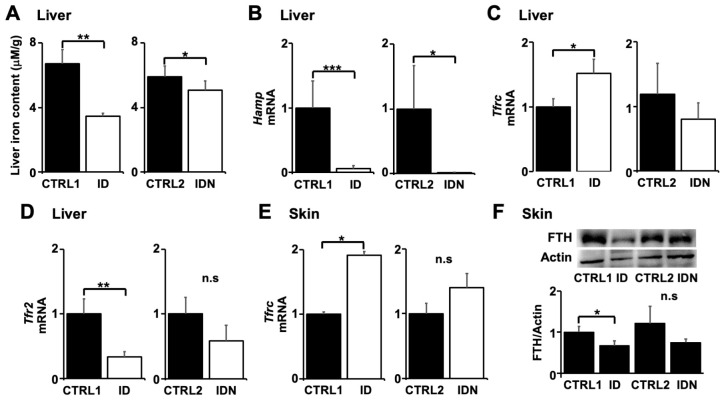
Early exposure to an iron-deficient diet altered iron content and expression of iron regulatory genes in liver and skin tissues. C57BL/6J pups born to iron-deficient dams were fed either an iron-sufficient (CTRL1) or iron-deficient diet (ID) for six weeks and then fed a normal iron-sufficient diet for two more weeks (CTRL2 and IDN). (**A**) The amount of iron in liver tissue was measured by Ferrozine assay (CTRL1, ID, CTRL2, IDN). The mRNA expression levels of (**B**) *Hamp*, (**C**) *Tfrc*, (**D**) *Tfr2* in liver tissue. The mRNA expression level of (**E**) *Tfrc* in skin tissue. The protein expression of (**F**) Ferritin heavy chain (FTH) in skin tissue from each group (CTRL1, ID, CTRL2, IDN) are represented. Values are mean ± SD. The differences between groups were tested using Student’s *t*-test. * *p* < 0.05; ** *p* < 0.01; *** *p* < 0.001; n.s., not statistically significant. All Western blot images within each panel were acquired under identical exposure conditions. Brightness and contrast adjustments were applied equally across the entire image.

Hepatic iron content in the ID group was 48.3% of the CTRL1 group, whereas after 2 weeks of supplementation hepatic iron content restored to approximately 86% of that observed in the CTRL2 group (Figure 2A). The expression of hepcidin in the liver, an iron-regulating hormone that inhibits systemic iron release, was downregulated in the ID group and remained low in the IDN group relative to controls (Figure 2B). The ID group exhibited a reduction in hepatic transferrin receptor 1 (*Tfrc*) mRNA levels, which was significantly reversed in the IDN group (Figure 2C). Additionally, liver mRNA levels of transferrin receptor 2 (*Tfr2*), an indicator of stored iron, were reduced in the ID group but normalized in the IDN group, compared with controls (Figure 2D). In skin tissue, the ID group demonstrated elevated *Tfrc* mRNA levels and decreased FTH1 protein levels relative to the CTRL1 group; however, these differences were not evident in the IDN group when compared to the CTRL2 group. (Figure 2E,F). These findings indicate that early iron deficiency led to reduced tissue iron levels and altered iron-related gene expression, which were restored by postnatal dietary iron repletion.

### 3.3. Iron Deficiency Impaired Wnt/β-Catenin Signaling in Hair Growth

**Figure 3:**
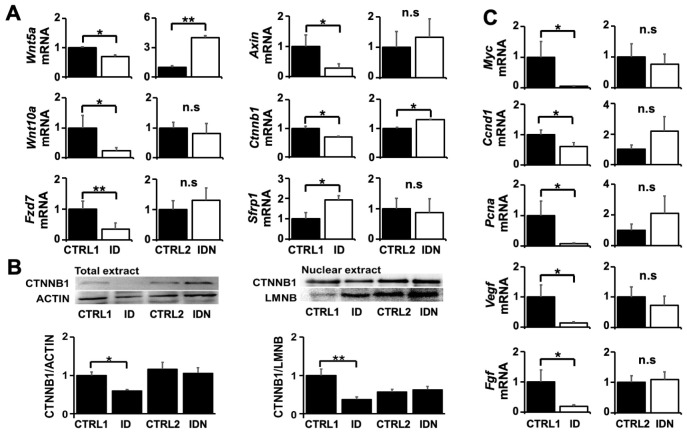
Early iron deficiency led to the inactivation of the Wnt/β-catenin pathway, a major mechanism of hair growth in mouse skin tissue. C57BL/6J pups born to iron-deficient mothers were fed either an iron-sufficient (CTRL1) or iron-deficient diet (ID) for six weeks and then fed a normal iron-sufficient diet for two more weeks (CTRL2 and IDN). (**A**) The mRNA expression of Wnt signaling pathway-related genes and (**B**) protein levels of total and nuclear β-catenin, (**C**) the mRNA expression of β-catenin target genes, measured in mouse skin tissue. Values are mean ± SD. The differences between groups were tested using Student’s *t*-test. * *p* < 0.05; ** *p* < 0.01; n.s., not statistically significant. All Western blot images within each panel were acquired under identical exposure conditions. Brightness and contrast adjustments were applied equally across the entire image.

The inhibitory effects of iron deficiency on hair growth were further evaluated through the Wnt/β-catenin signaling pathway because this pathway is crucial for the regulation of hair follicle development [17,18]. The ID group exhibited reduced mRNA expression of *Wnt5a*, *Wnt10a*, *Fzd7* (a WNT receptor), *Axin1*, and *Ctnnb1* compared to the CTRL1 group, with these reductions being reversed in the IDN group (Figure 3A). Furthermore, the reduction in total and nuclear CTNNB1 protein levels, as well as the mRNA levels of its target genes—including *Myc*, cyclin D1 (*Ccnd1*), proliferating cell nuclear antigen (*Pcna*), vascular endothelial growth factor (*Vegf*), and fibroblast growth factor (*Fgf*) was observed in the ID group but not in the IDN group (Figure 3B,C). Elevated mRNA levels of *Sfrp1*, a WNT antagonist, were also noted in the ID group, whereas these levels were not elevated in the IDN group (Figure 3A). These findings suggest that early iron deficiency was associated with impaired Wnt/β-catenin-mediated follicular signaling, which was restored following dietary iron supplementation under the conditions of this study.

### 3.4. Iron Deficiency Activated SMAD-Dependent TGF-β Signaling and Apoptosis in the Skin

**Figure 4:**
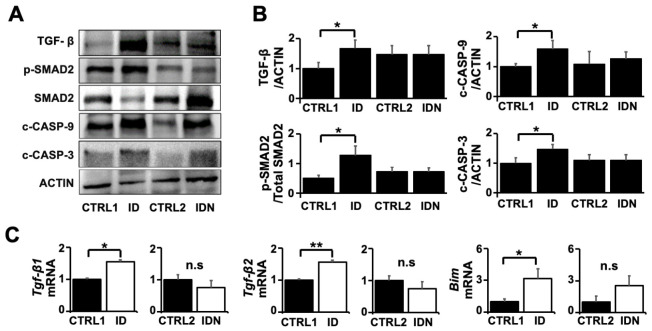
Early iron deficiency activated cell death and apoptotic signaling pathways in mouse skin tissue. C57BL/6J pups born to iron-deficient mothers were fed either an iron-sufficient (CTRL1) or iron-deficient diet (ID) for six weeks and then fed a normal iron-sufficient diet for two more weeks (CTRL2 and IDN). (**A**,**B**) Protein levels of TGF-β, and phosphorylation levels of SMAD, cleaved caspase-9 and -3, measured in mouse skin tissues by Western blot analysis. (**C**) The mRNA expression of *Tgf-β1* and *2*, *Bim* in mouse skin tissue. Values are mean ± SD. The differences between groups were tested using Student’s *t*-test. * *p* < 0.05; ** *p* < 0.01; n.s., not statistically significant. All Western blot images within each panel were acquired under identical exposure conditions. Brightness and contrast adjustments were applied equally across the entire image.

TGF-β signaling is known to suppress hair follicle stem cell proliferation and promote the transition from anagen to catagen [19]. The lack of hair growth in the ID group was further analyzed through the TGF-β pathway. Activation of TGF-β1 signaling, as evidenced by phosphorylation of SMAD, was observed in the ID group but not in the IDN group, compared to the control groups (Figure 4A,B). Additionally, the cleaved forms of CASP9 and CASP3, markers of apoptosis, were significantly elevated in the ID group but not in the IDN group (Figure 4A,B). The mRNA levels of *Tgfb1*, *Tgfb2*, and *Bim*, a pro-apoptotic gene, were upregulated in the ID group compared to the CTRL1 group but showed no such increase in the IDN group relative to the CTRL2 group (Figure 4C). Our data suggest that early iron deficiency was associated with increased TGF-β-mediated apoptotic signaling in skin tissue, which was attenuated following postnatal iron supplementation.

### 3.5. Oxidative Stress as a Mediator of Aberrant Signaling Pathways in Iron Deficiency-Related Hair Loss

**Figure 5:**
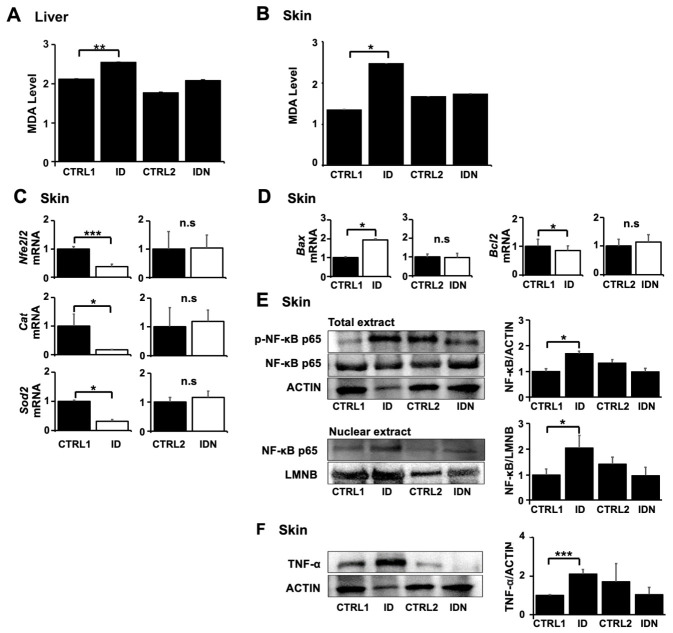
Early iron deficiency increased oxidative stress and the NF-κB pathway in the skin, but the changes were abolished by iron supplementation. C57BL/6J pups born to iron-deficient mothers were fed either an iron-sufficient (CTRL1) or iron-deficient diet (ID) for six weeks and then fed a normal iron-sufficient diet for two more weeks (CTRL2 and IDN). (**A**) MDA levels measured in mouse liver tissue and (**B**) skin tissue. Also, the mRNA expression of (**C**) antioxidant markers (Nfe2l2) and mitochondrial antioxidant markers (Cat, Sod2), and (**D**) Bax and Bcl2 measured in mouse skin tissues were confirmed by qPCR analysis. Western blot analysis of (**E**) total and nuclear expression of the transcription factor NF-κB and (**F**) expression of TNF-α in mouse skin tissue. Values are mean ± SD. The differences between groups were tested using Student’s *t*-test. * *p* < 0.05; ** *p* < 0.01; *** *p* < 0.001; n.s., not statistically significant. All Western blot images within each panel were acquired under identical exposure conditions. Brightness and contrast adjustments were applied equally across the entire image.

Reactive oxygen species (ROS) are often linked to disrupted iron regulation due to iron’s redox potential [20,21]. In addition, excessive ROS can have detrimental effects by promoting premature catagen entry and apoptosis-driven follicle regression, ultimately reducing the potential for hair regrowth [22,23]. To investigate the underlying mechanisms of iron deficiency in relation to aberrant signaling pathways, we explored the role of reactive oxygen species (ROS). We first assessed hepatic malondialdehyde (MDA) levels as a marker of lipid peroxidation. The ID group exhibited significantly higher hepatic MDA levels compared to the CTRL1 group, whereas the levels in the IDN and CTRL2 groups were comparable (Figure 5A). Skin MDA levels showed a similar pattern. The ID group exhibited significantly higher skin MDA levels than the CTRL1 group, whereas no significant difference was observed between the CTRL2 and IDN groups (Figure 5B). In skin tissue, alterations in mRNA expression of nuclear factor erythroid 2-related factor 2 (*Nrf2*), an antioxidant transcription factor known to upregulate *Bcl2* [24] and mitochondrial antioxidant enzymes such as catalase (*Cat*) and superoxide dismutase 2 (*Sod2*) and downregulate *Bax* [24], were observed in the ID group but not in the IDN group relative to their respective controls (Figure 5C). Moreover, increased levels of phosphorylated and nuclear-localized NFKB1 were detected in the skin tissue of the ID group, while these levels were normalized in the IDN group compared to the controls (Figure 5E). The pro-inflammatory cytokine *Tnf*, an NFKB1 target known to be upregulated in response to oxidative stress [25], was also elevated in the skin tissue of the ID group but not in the IDN group compared to their respective controls (Figure 5F). Additionally, the ID group demonstrated reduced *Bcl2* mRNA levels and elevated *Bax* mRNA levels in skin tissue compared to the CTRL1 group, while no such differences were noted between the IDN and CTRL2 groups (Figure 5D). Our findings indicate that early iron deficiency might have induced oxidative stress, potentially via the downregulation of *Nrf2* and enhancing the NFKB1-mediated response. These results suggest that ROS may be involved in the detrimental effects associated with iron deficiency.

### 3.6. Role of Oxidative Stress in Iron Deficiency-Mediated Disruption of Wnt/β-Catenin Signaling in HFDPCs

**Figure 6:**
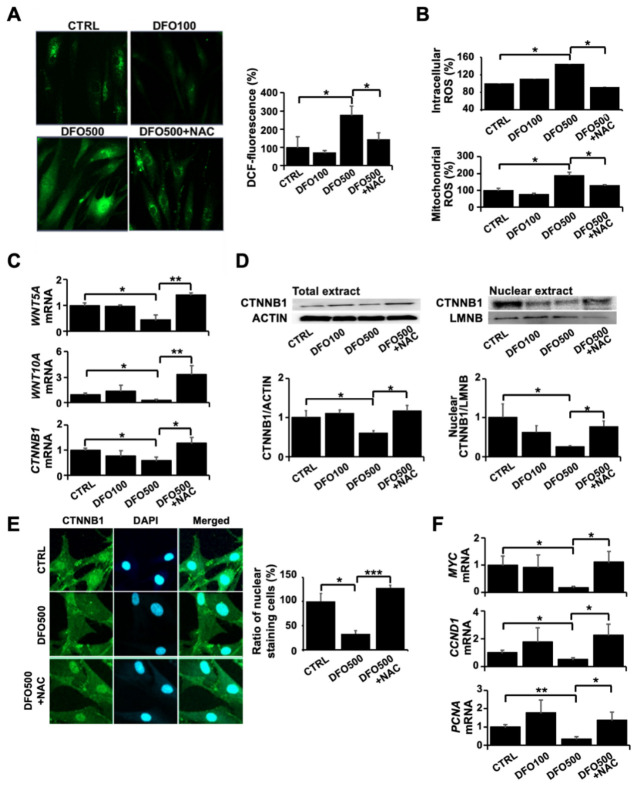
DFO, an iron chelator, induced cell cycle arrest and oxidative stress but had no effect on mitochondrial function in HFDPCs. HFDPCs were treated with either water (control) or different concentrations of DFO (100 and 500 μM) for 24 h. Cells belonging to the *N*-acetylcysteine (NAC) group were treated with 100 μM NAC for 1h before treatment with 500 μM DFO. (**A**) DCF-DA staining was performed to analyze ROS using fluorescence microscopy. (**B**) Levels of intracellular and mitochondrial ROS were determined using DCF-DA and MitoSOX assays, respectively. (**C**) mRNA expression of Wnt/β-catenin pathway-related genes measured using qPCR, and (**D**) the protein expression of β-catenin in total protein and nuclei measured using Western blot analysis. (**E**) β-catenin was probed by anti-β-catenin and FITC-conjugated secondary antibodies in fixed HFDPCs. (**F**) mRNA expression of β-catenin target genes was measured using qPCR. Student’s *t*-test was used to test for differences between groups. * *p* < 0.05; ** *p* < 0.01; *** *p* < 0.001. All Western blot images within each panel were acquired under identical exposure conditions. Brightness and contrast adjustments were applied equally across the entire image.

To explore whether the effects we observed might be ROS-dependent, we carried out an in vitro experiment using primary human follicle dermal papilla cells (HFDPCs). We assessed if pretreatment of the hair follicle cells with *N*-acetylcysteine (NAC), an antioxidant, before exposing them to deferoxamine (DFO), an iron chelator, altered ROS levels. DFO treatment significantly elevated both cellular and mitochondrial ROS levels (Figure 6A,B) in the absence of NAC pre-treatment, and lead to increased mRNA expression of *TFRC* and decreased FTH1 levels, as expected, confirming an iron-deficient cellular state (Supplementary Figure S4. Pre-treatment with NAC effectively abrogated this DFO-mediated ROS increase (Figure 6A,B). Moreover, DFO exposure reduced mRNA levels of *WNT5A*, *WNT10A*, and *CTNNB1* (Figure 6C), decreased nuclear CTNNB1 levels (Figure 6D,E), and suppressed the expression of *CTNNB1* target genes, including *MYC*, *CCND1*, and *PCNA* (Figure 6F). NAC treatment attenuated the DFO-induced suppression of Wnt/β-catenin signaling (Figure 6C–F), suggesting that ROS may contribute to the inhibitory effects of iron deficiency on hair follicle-related signaling in HFDPCs.

### 3.7. Role of Oxidative Stress in Iron Deficiency-Induced Apoptosis and NF-κB Signaling in HFDPCs

**Figure 7:**
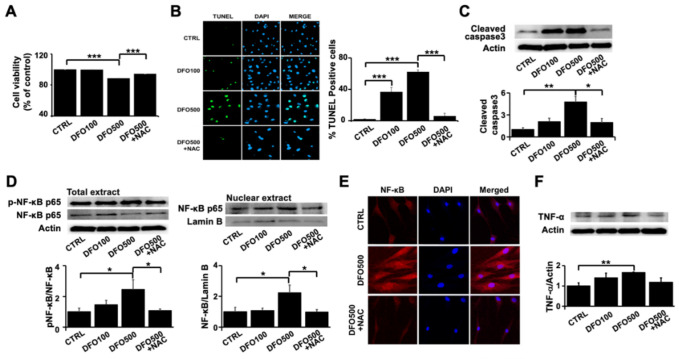
NAC, an antioxidant chemical, abolished DFO-induced apoptosis and NF-κB activity in HFDPCs. HFDPCs were treated with either water (control) or different concentrations of DFO (100 and 500 μM) for 24 h. Cells belonging to the *N*-acetylcysteine (NAC) group were treated with 100 μM NAC for 1 h before treatment with 500 μM DFO. (**A**) Cell viability was measured using the MTT assay and (**B**) apoptotic cells were determined using the TUNEL assay. (**C**) Representative blot image of cleaved caspase-3 protein expression after DFO and/or NAC treatment in HFDPCs. (**D**) Protein levels of NF-κB p65 and phospho NF-κB p65 in total and nucleus of HFDPCs were measured, and the representative blot images were presented. (**E**) NF-κB/p65 subunits were probed by the anti-NF-κB polyclonal primary antibody and rhodamine-conjugated secondary antibody in fixed HFDPCs. Nuclei were stained with DAPI, and images were captured using confocal microscopy. (**F**) Protein expression levels of TNF-α, *p*-SAMD2 and SMAD2 in HFDPCs were measured. For Western blot analysis, either actin or lamin B was used as a loading control. * *p* < 0.05; ** *p* < 0.01; *** *p* < 0.001. All Western blot images within each panel were acquired under identical exposure conditions. Brightness and contrast adjustments were applied equally across the entire image.

Pretreatment with NAC attenuated the DFO-induced increase in MTT-positive and TUNEL-positive HFDPCs, indicating reduced inhibition of cell proliferation and decreased apoptosis (Figure 7A,B). Congruent with these findings, NAC also mitigated the DFO-mediated elevation of cleaved CASP3 levels, a key marker of apoptosis (Figure 7C). Furthermore, NAC abolished DFO-induced phosphorylation and nuclear translocation of NFKB1 (Figure 7D,E), and significantly lowered the increase in TNF levels and phosphorylation of SMAD2 induced by DFO (Figure 7F). These findings demonstrate that NAC-mediated ROS reduction reduces iron deficiency-associated cell death and modulates related apoptotic and inflammatory signaling pathways, including CASP3 and NFKB1 in the HFDPC model.

## 4. Discussion

Our study provided evidence that the failure of hair growth associated with early iron deficiency is accompanied by elevated ROS levels, reduced Wnt/β-catenin signaling, and increased apoptotic markers in vivo. Postnatal dietary iron supplementation restored visible hair growth and largely reversed these molecular alterations. In iron-deficient HFDPCs, ROS reduction was sufficient to restore several hair follicle-related signaling changes, and mitigated DFO-associated cell death, supporting a possible role for ROS in iron deficiency-associated cellular dysfunction. Together these findings suggest that iron deficiency-associated alterations in redox homeostasis are linked to the hairless phenotype, which remains reversible by dietary iron repletion. Although further research is warranted to definitively establish direct in vivo causality, our data suggest that early iron deficiency is associated with altered hair cycle distribution, characterized by reduced anagen and increased telogen follicles, while preserving the potential for subsequent hair regrowth after iron replenishment.

A causal relation between low iron availability and impaired hair growth had been suggested by previous experimental studies. Severe systemic iron deprivation in liver specific hepcidin transgenic mice resulted in microcytic hypochromic iron deficiency anemia and produced pups with pale skin, smaller body size, and truncal hair loss [9]. Similarly, genetically modified mice lacking *Tmprss6*, which encodes matriptase-2, a transmembrane serine protease that suppresses hepcidin expression, developed severe microcytic anemia and regional truncal alopecia [26]. Hair loss has also been observed in pups born to hephaestin knockout or sex-linked anemia (*sla*) dams, in which impaired intestinal iron transport leads to iron-deficient milk production [11,12]. Consistent with these reports, pups born to dams fed an iron-deficient diet and maintained on the same diet after weaning showed reduced liver iron content, lowered body weight, and truncal hair loss in our study. Although iron-deficiency anemia was not directly assessed, reduced hepatic iron content together with altered iron-related gene expression supports the presence of systemic iron deprivation.

Hair follicles are highly proliferative organs and hair growth may be particularly sensitive to reduced iron availability, even in the absence of overt changes in systemic hematological indices. Transgenic mice overexpressing human H-ferritin exhibited smaller body size and transient truncal hair loss without developing anemia, as indicated by the absence of significant changes in hemoglobin levels or red blood cell counts [27]. Mice with macrophage-specific deletion of ferroportin-1 developed epithelial iron deprivation, transient truncal hair loss, and dilated hair follicles at birth despite the absence of anemia [10], highlighting the importance of local iron availability in hair follicle development. In addition, reduced hepatic iron stores in *Fpn*^null/+^ animals were reported to cause a mild defect in erythropoiesis without anemia [28].

Clinical observations further supported the possibility that hair growth may be affected by reduced iron availability even when hematological indices remain within the normal range. A recent human study reported that iron supplementation improved female alopecia in individuals with hemoglobin levels within the normal range [14]. In that study, serum ferritin levels 40 to 60 ng/mL were suggested to be favorable for hair growth [14], a range higher than the threshold commonly used to define iron-deficiency anemia, typically below 15 to 30 ng/mL [29]. Consistently, Deloche et al. reported that 59% of women with excessive hair loss had hair loss and serum ferritin levels below 40 ng/mL [30]. Together, these findings support the possibility that the level of iron availability required for optimal hair growth and/or skin homeostasis may be higher than that required to prevent anemia, although this concept warrants further investigation [31].

The hair follicle is a dynamic organ whose structural homeostasis depends on its cyclic progression through distinct phases, including anagen (growth), catagen (transition), telogen (resting), and exogen (shedding) [32]. Various intrinsic and extrinsic factors, including oxidative stress, inflammation, diet, and psychological stress, can influence hair cycle progression, particularly the transition from anagen to catagen [32,33]. The requirement for iron in hair growth has been supported by previous studies using genetically modified animals with defects in dietary iron absorption, transport, or utilization. For instance, H-ferritin overexpressing mice delayed the entry of hair follicles into anagen [27], whereas macrophage-specific deletion of ferroportin failed to maintain the proliferative state of hair follicles [10]. A potential direct effect of dietary iron deficiency on hair growth was also demonstrated by a study showing that a low maternal iron diet caused hair loss in pups under IL10-deficiency-associated immune dysregulation [34].

The multifaceted role of iron in maintaining hair follicle health may be related, at least in part, to its requirement for rapid cellular proliferation, particularly during the anagen phase of the hair cycle. Iron plays as a cofactor for DNA polymerases, which are crucial for cell proliferation, as well as it may influence hair cycle regulation by modulating the expression of key genes such as CDC2, a gene for cell cycle progression [35], NDRG1, a stress response gene [36] and ALAD, a gene for heme production [37] in the bulge region of the human hair follicle. In our study histological analysis showed a shift in hair cycle distribution from anagen to telogen follicles in the ID group, although catagen follicles were not captured at our evaluation. This absence of detectable catagen follicles may be attributable, at least in part, to the brief and transitory nature of the catagen phase in the rodent hair cycle, which typically spans only a few days [15]. Thus the observed increase in telogen follicles may reflect impaired maintenance of the anagen phase and/or delayed re-entry into anagen. Together with increased caspase-3 expression, iron deficiency was associated with reduced follicular growth activity and increased apoptotic signaling, which might have contributed to altered hair cycle distribution without significant changes in total follicle density.

The reversibility of truncal hair loss observed in our study is consistent with previous reports. Truncal hair loss associated with increased hepcidin expression was reversed by iron supplementation [9], and hair loss in pups born to IL10^−/−^ dams fed an iron deficient diet was resolved after weaning [34]. Recalcati, Gammella et al. reported transient alopecia in genetically modified mice with disrupted local iron homeostasis [10]. Our data also indicated that early iron deficiency did not result in irreversible hair loss under the conditions of our study, as hair regrowth and the expression of molecular markers associated with hair follicle growth were restored following iron replenishment. Oxidative stress-related alterations observed in iron-deficient pups, including elevated hepatic lipid peroxidation and changes in antioxidant enzyme expression in both liver and skin tissues, were no longer evident after iron supplementation. Together, these findings may suggest that sufficient and sustained iron availability is important for maintaining normal skin and hair follicle development.

Our data may suggest that the amount of iron required to support hair regrowth does not necessarily reflect complete systemic iron restoration. Although truncal hair was fully regrown after two weeks of iron supplementation, hepatic iron levels and hepcidin expression remained lower than those in the iron sufficient control group. In contrast, the short-term iron supplementation was sufficient to normalize tissue level indicators, such as skin ferritin expression and hepatic transferrin receptor 2 (TfR2) expression. However, because hematological parameters, such as hemoglobin levels and hematocrit, were not assessed, we cannot fully distinguish whether the observed hair recovery reflects localized tissue sufficiency or a degree of systemic iron replenishment sufficient to support hematopoiesis and other iron-dependent function. The apparent discrepancy between hair regrowth and incomplete recovery of hepatic iron stores may have reflected a temporal hierarchy of iron redistribution during early iron refeeding, in which skin homeostasis and hair follicle growth recovered more rapidly than hepatic iron storage. Alternatively, the skin and hair follicle may have required a smaller iron pool for functional recovery than the liver, allowing these tissues to respond more rapidly to short-term iron Nevertheless, the incomplete recovery of hepatic hepcidin expression in these animals was consistent with the role of hepcidin as a systemic sensor reflecting excess body iron stores.

Hair follicle morphogenesis begins in the early embryonic stage with the condensation of dermal papillary fibroblasts and the thickening of the epidermis to form the hair germ. Subsequent rapid epithelial cell proliferation contributes to the formation of bulb-like structures in the hair peg, eventually developing the hair cortex and shaft [38]. A key signal for this induction is the Wnt/β-catenin pathway. In our study, iron deficiency in both in vivo and in vitro studies was associated with disrupted Wnt/β-catenin pathway and altered expression of growth- and cell cycle-related genes, including EGF, VEGFA, IGF, FGF1, Myc [39], and CCND1 [40]. In addition, iron deficiency in HFDPCs increased the expression of the genes associated with G0/G1 phase cell cycle arrest and apoptotic cell death (Supplementary Figure S5). Although our in vivo data demonstrate an association rather than direct casualty, our in vitro NAC experiment provides supportive mechanistic evidence. In iron deficient HFDPCs, ROS reduction restored Wnt/β-catenin signaling and reduced apoptosis, suggesting that iron deficiency-associated oxidative stress may contribute to the suppression of that Wnt/β-catenin signals and impaired hair follicle growth. Nevertheless, because a direct causal relationship between excessive ROS and defective Wnt/β-catenin signaling was not established in vivo, future studies using ROS-scavengers approaches, such as dietary or pharmacological antioxidants, are warranted to determine whether ROS acts as an upstream mediator of impaired Wn/β-catenin signaling and iron deficiency-induced hair loss. Our findings aligned with previous studies testing chemicals or extracts for their effects on alopecia, which reported that decreased Wnt/β-catenin signaling correlates with decreased hair cell growth and arrest of hair cell cycle [41,42]. Therefore, iron deficiency-induced hair loss may have been closely linked to suppression of Wnt/β-catenin-associated proliferative signaling and increased markers of cell cycle arrest and apoptosis.

The transition from anagen to catagen can be driven by the activation of caspases. Signals driving the onset of catagen, whether intrinsic or extrinsic, converge on the activation of caspase-3, leading to apoptotic cell death characterized by DNA fragmentation [43]. Previous studies have shown that TGF-β treatment in human hair follicles induces catagen-like changes by activating these caspase signals [44]. Specifically, TGF-β1 is known to promote catagen [19], while TGF-β2 suppresses hair elongation [45]. In contrast, TGF-β3 is primarily associated with wound healing [46]. In transgenic mice overexpressing Smad-2, activation of TGF-β/SMAD-2 signaling led to delayed hair growth [47]. Although the precise molecular mechanisms underlying iron deficiency-induced hairlessness require further investigation, iron deficiency-associated activation of TGF-β/SMAD-2 signaling may have contributed to, rather than solely accounted for, the apoptotic and catagen-like changes observed in hair follicles.

ROS appear to play a dual role in hair development. While transient or local levels of ROS can act as signaling molecules required for hair follicle formation and regeneration [48], excessive ROS may induce oxidative damage and hair follicle degeneration [49]. The necessity of ROS has been highlighted during hair follicle differentiation, when a metabolic switch from glycolysis to oxidative phosphorylation occurs during the transition from telogen to anagen. In an in vivo animal study, an increased mitochondrial activity was observed during hair follicle differentiation [50] but interestingly, this increase in mitochondrial activity was not accompanied by a significant elevation in ROS levels, which the authors attributed to the concurrent up-regulation of SOD2 [50]. In addition, a mouse model with deletion of the mitochondrial transcription factor *Tfam* demonstrated that mitochondrial ROS was required for β-catenin signaling and hair follicle development [51]. An ex vivo study using human hair follicles also showed the necessity of endogenous ROS production for the activation of β-catenin signals and hair growth [52].

In contrast, excessive oxidative stress had been closely associated with impaired hair follicle maintenance and alopecia. In humans, alopecia has been linked to oxidative stress-mediated cellular senescence [22]. Oxidative stress driven by Foxp1 deficiency or impaired DNA repair led to premature catagen transition and follicle degeneration [23]. Delayed hair growth was observed in offspring born to albino rats that received iron chelate injections and exhibited excessive oxidative stress. These injections also caused increased pregnancy failure and defects in postnatal development [53]. Dietary iron deficiency from the fetal developmental stage until adulthood had similarly been shown to increase oxidative stress in organs including brain, blood, and liver, while depleting antioxidant defenses such as SOD and glutathione peroxidase in rats [54]. Conversely, application of antioxidant-conjugated gelatin-containing hydrogel in an animal model with a full-thickness wound scavenged ROS and promoted new hair follicle formation [55]. Although we were not able to directly measure ROS levels in vivo, increased skin lipid peroxidation together with suppressed antioxidant gene expression under iron-deficient conditions may have indicated a redox imbalance, which could have hindered hair growth in mice. This was further supported by our in vitro findings, in which iron chelation clearly increased cellular and mitochondrial ROS levels. In addition, previous studies have shown that iron deficiency anemia can increase autoxidation in red blood cells and thereby contribute to systemic oxidative stress [56]. Although anemia was not assessed in our animals, systemic oxidative stress associated with iron deficiency might also have contributed to the hair growth defects observed in our study. Taken together, iron deficiency may be associated with elevated ROS production and disrupted redox homeostasis, thereby hindering normal hair growth.

Although the source of the elevated ROS in iron-deficient mouse skin tissue remains to be definitively characterized, our in vitro assays showed increased mitochondria ROS signals in iron deficient HFDPCs. However despite the increase in mitochondrial ROS, mitochondrial membrane potential (MMP) and ATP production showed no significant alterations (Supplementary Figure S6). The absence of detectable changes in MMP and ATP levels may suggest that the degree of iron deficiency in our study was sufficient to increase mitochondrial ROS production but not severe enough to compromise overall ATP synthesis.

Previous studies have reported that iron deficiency can impair the mitochondrial electron transport chain (ETC), thereby exacerbating mitochondrial oxidative stress. Iron deficiency was shown to disrupt the ETC by inhibiting the maturation of iron-sulfur (Fe-S) cluster proteins and inducing mitochondrial DNA damage [57]. In addition, iron deficiency suppresses mitochondrial complex I activity even in the absence of anemia [58]. It should also be noted that reduced mitochondrial respiratory activity does not necessarily result in decreased MMP or ATP levels, because cells can maintain MMP through the reverse activity of ATP synthase, fueled by glycolytic ATP [59]. Thus, preserved MMP and ATP levels in our HFDPCs did not exclude the possibility of mitochondrial dysfunction or altered electron flow. The source of mitochondrial ROS under iron deficient conditions may also involve impaired activity of iron-containing mitochondrial enzymes, such as aconitase 2, which is sensitive to Fe-S cluster homeostasis [60]. Taken together, iron deficiency-induced mitochondrial ROS in HFDPCs may have arisen from subtle alterations in ETC function and/or Fe-S cluster-dependent mitochondrial enzymes, even before overt impairment of MMP or ATP production becomes evident.

Previous studies have shown that iron chelation in breast cell lines increases mitochondrial ROS production, which is concomitant with the upregulation of TGF-β and NF-κB signaling [61]. Data from patients with androgenetic alopecia exhibited lower antioxidant levels alongside elevated serum TGF-β1, NF-κB, and TNF-α [61]. Furthermore, transgenic mice with constitutive NF-κB activation show significant growth retardation and impaired hair formation [62]. Conversely, the inactivation of NF-κB signaling such as through DKK1 knockout has been shown to restore hair growth by upregulating Wnt-related genes [63]. Similarly, in our in vitro experiments using HFDPCs, antioxidant treatment reduced iron deficiency-induced ROS accumulation and partially restored expression of several hair-related genes. These findings were consistent with our in vivo observations showing impaired hair growth and altered expression of signaling molecules related to Wnt/β-catenin, TGF-β1, NF-κB, and TNF-α. Moreover, an iron-supplemented diet was associated with the restoration of these molecular changes and visible hair regrowth.

The association between maternal iron deficiency and adverse infant outcomes has been widely reported [64]. The timing of iron deficiency may be particularly important. For instance, infants born to non-anemic women with depleted iron stores during the first trimester showed lower birth weights despite subsequent daily iron supplementation until delivery [65]. In the present study, one-week prenatal exposure to maternal iron deficiency was used as an experimental window corresponding approximately to an early gestational period in humans. Pups exposed to maternal iron deficiency prenatally and maintained on an iron-deficient diet for six weeks after birth exhibited significantly lower body weight than control pups. Notably, while two weeks of postnatal iron supplementation was sufficient to restore visible hair growth, it did not fully reverse the reduction in body weight under the conditions of this study. This discrepancy may suggest that the relatively short duration of iron supplementation was not sufficient to correct all developmental effects associated with early life iron deficiency. Overall, our findings support the importance of adequate maternal iron status during early development, while further studies are needed to determine whether longer or earlier iron repletion can fully restore growth-related outcomes.

Despite these findings, several limitations should be considered. Because dams were fed an iron-deficient diet during both gestation and lactation, we could not distinguish whether offspring iron deficiency primarily originated during fetal development or from iron-deficient milk intake during suckling. In addition, the relatively small number of animals in each experimental group may have limited the statistical power of the study. Hematologic parameters were also not assessed because of the limited blood volume obtainable from offspring mice, making it difficult to distinguish the effects of systemic anemia from those of tissue iron deficiency. Although our in vitro NAC experiments suggest that ROS accumulation is involved in the suppression of Wnt/β-catenin signaling and apoptosis in iron-deficient HFDPCs, this mechanistic link was not directly tested in vivo. Therefore, future studies using in vivo antioxidant interventions are needed to determine whether ROS scavenging can restore Wnt/β-catenin signaling and rescue hair growth in iron-deficient pups.

Furthermore, our findings should be extrapolated to human clinical settings with caution, given physiological differences between mice and humans. Whereas human hair growth occurs in an asynchronous and mosaic pattern, mice exhibit a synchronized hair cycle during early postnatal life, which may increase the apparent phenotypic impact of signaling disruptions, resulting in a more visually detectable hair growth phenotype than would be expected in humans. Nevertheless, our findings suggest that insufficient iron availability during early life is associated with impaired hair growth and provide evidence supporting a possible mechanistic role for ROS in the suppression of Wnt/β-catenin signaling during hair development under iron-deficient conditions.

## 5. Conclusions

Overall, our data suggest that early life iron deficiency disrupted redox homeostasis and was accompanied by suppression of Wnt/β-catenin signaling and activation of TGF-β and NF-κB pathways, which might have contributed to apoptotic loss of hair follicle cells. Notably, postnatal iron supplementation largely reversed these molecular alterations and restored hair growth, suggesting that the detrimental effects of early iron deficiency on hair follicle function may have been at least partially reversible under the conditions of this study. Future studies using in vivo antioxidant interventions, hematological assessments, tissue-specific iron tracking, and stage-specific iron deficiency models, and larger cohorts are needed to resolve the causal role of ROS and to distinguish the relative contributions of anemia, local tissue iron deficiency, prenatal iron deficiency, and postnatal iron deficiency during suckling.

## Data Availability

Data described in the manuscript will be made available upon request and approval of Principal Investigator (S.-M.L.).

## Supplementary Materials

The following supporting information can be downloaded at: https://www.mdpi.com/article/10.3390/nu18142321/s1, Table S1: Composition of different iron concentrated diets; Table S2: List of PCR primers used for the experiments; Figure S1: Gross phenotype of mice in each experimental group; Figure S2: Comparison of mitochondrial DNA (mtDNA) content among the experimental groups. Bars represent mean ± standard deviation; Figure S3: Early iron deficiency did not alter mitochondrial ROS production in mice skin. Bars represent mean ± standard deviation; Figure S4: DFO treatment increased Tfr1 mRNA expression and decreased FTH1 protein expression. Bars represent mean ± standard deviation. * *p* < 0.05; ** *p* < 0.01; *** *p* < 0.001; Figure S5: Effects of DFO treatment on cell death and cell cycle arrest in HFDPC. Results of analysis of (A) Cell viability, (B and C) FACS. Statistical significant were analyzed by student *t*-test (one tail). Bars represent mean ± standard deviation. * *p* < 0.05, ** *p* < 0.01. Figure S6: DFO did not affect mitochondrial dysfunction in HFDPCs. The result of (A) ATP level and (B) MMP level to measure mitochondrial dysfunction. Bars represent mean ± standard deviation.

## Abbreviations

The following abbreviations are used in this manuscript:

B2M	beta-2-microglobulin
CAT	catalase
CCND1	cyclin D1
COXII	cytochrome c oxidase II
CTRL	control
Ct	cycle threshold
DFO	deferoxamine
FGF	fibroblast growth factor
HFDPCs	human follicle dermal papilla cells
ID	iron-deficient diet;
IDN	normal diet after iron deficiency
MMP	mitochondrial membrane potential
mtDNA	mitochondrial DNA
TfR1	transferrin receptor 1
TUNEL	terminal deoxynucleotidyl transferase dUTP nick end labeling
