# Supplementary figures and images for: Iron Deficiency-Induced Hair Loss Is Associated with ROS-Mediated Disruption of Wnt/β-Catenin Signaling
Source: Nutrients. 2026 Jul 15;18(14):2321. doi: 10.3390/nu18142321 (PMC13414816; doi:10.3390/nu18142321)

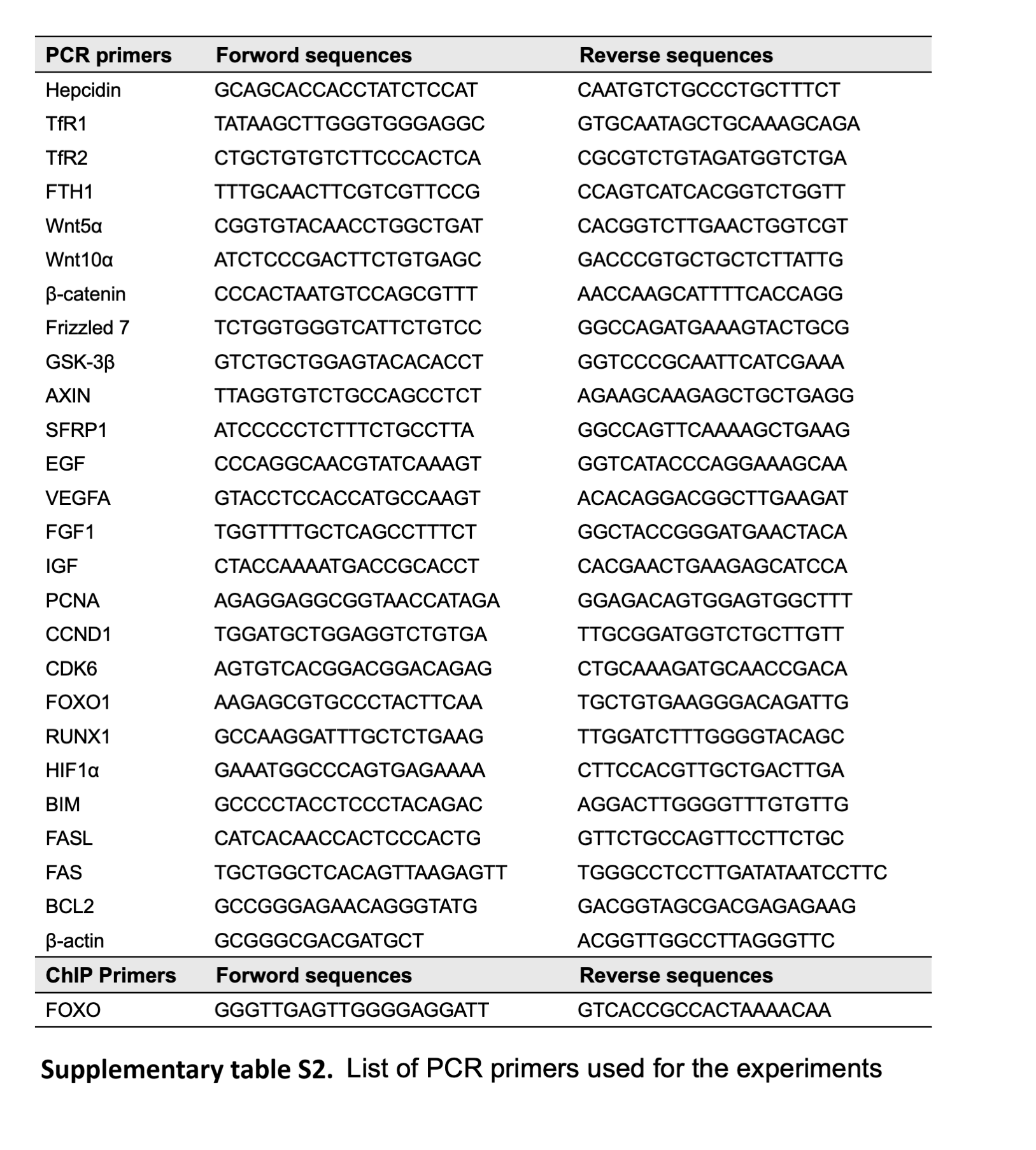

Supplement: Supplementary file 1 [file nutrients-18-02321-s001.zip › Supplementary table S2.tiff]

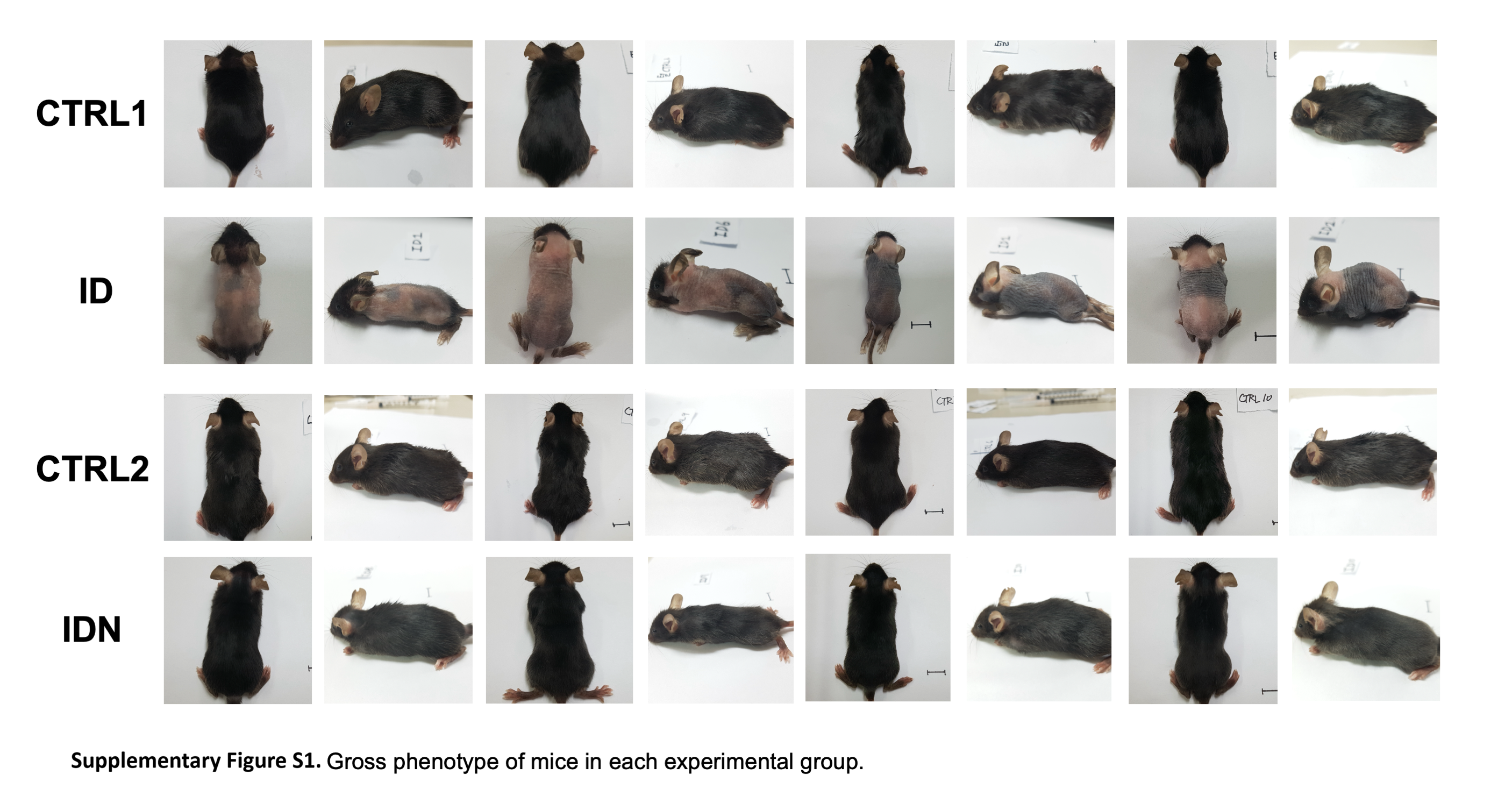

Supplement: Supplementary file 1 [file nutrients-18-02321-s001.zip › Supplementary figure S1.tiff]

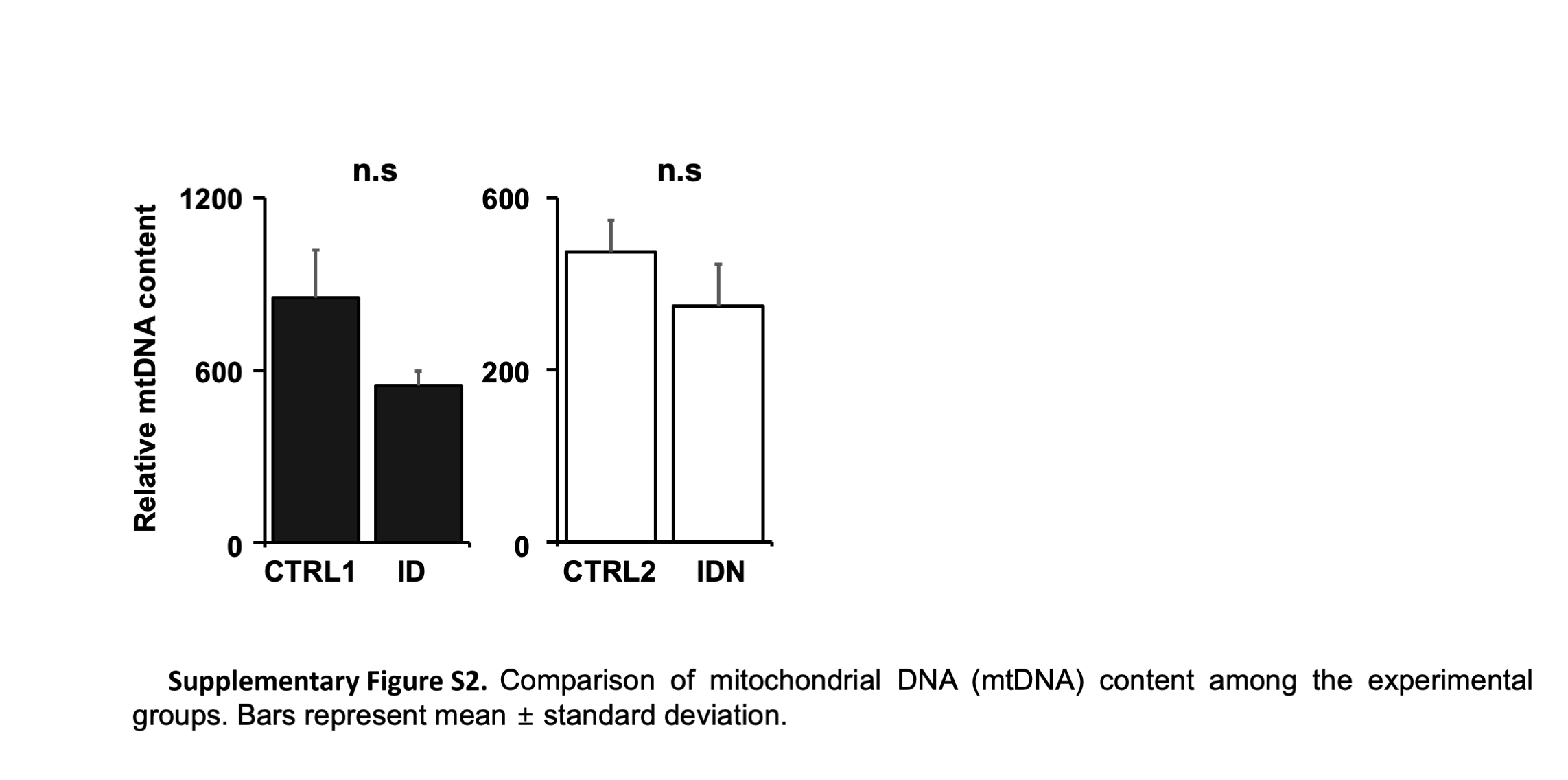

Supplement: Supplementary file 1 [file nutrients-18-02321-s001.zip › Supplementary figure S2.tiff]

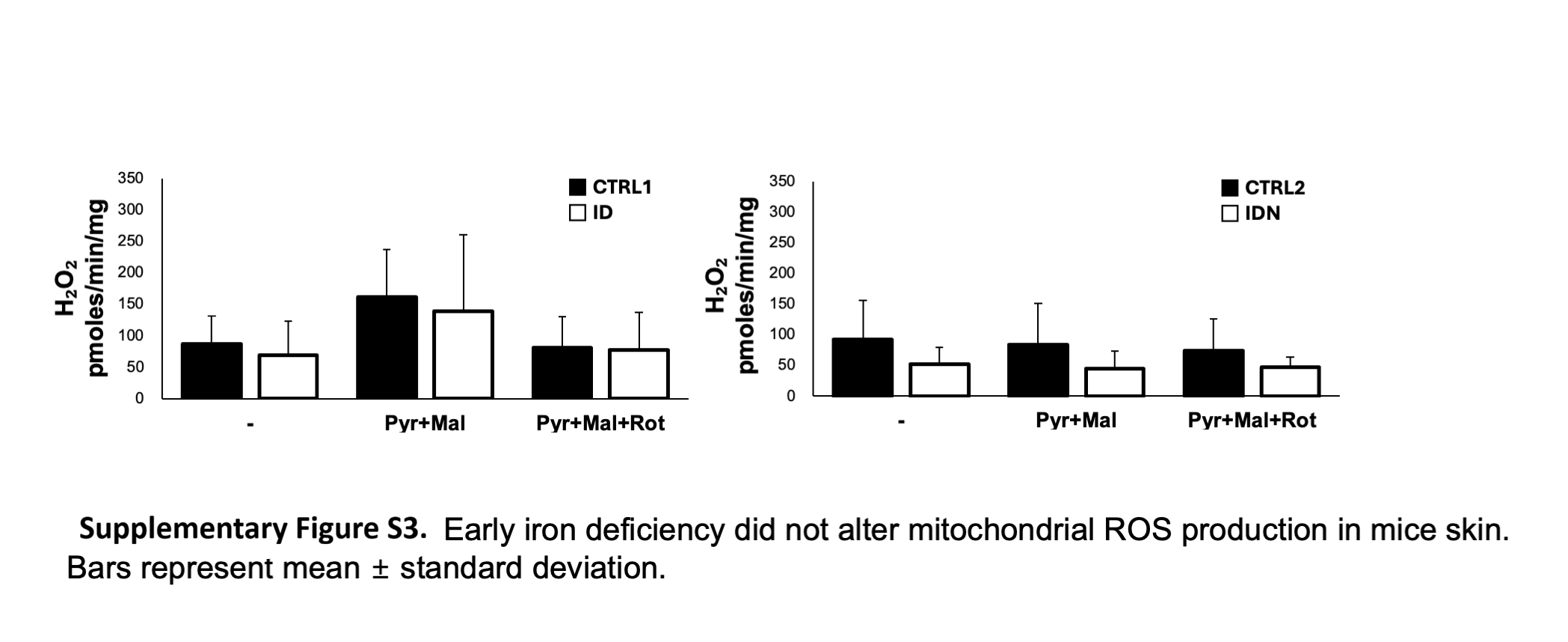

Supplement: Supplementary file 1 [file nutrients-18-02321-s001.zip › Supplementary figure S3.tiff]

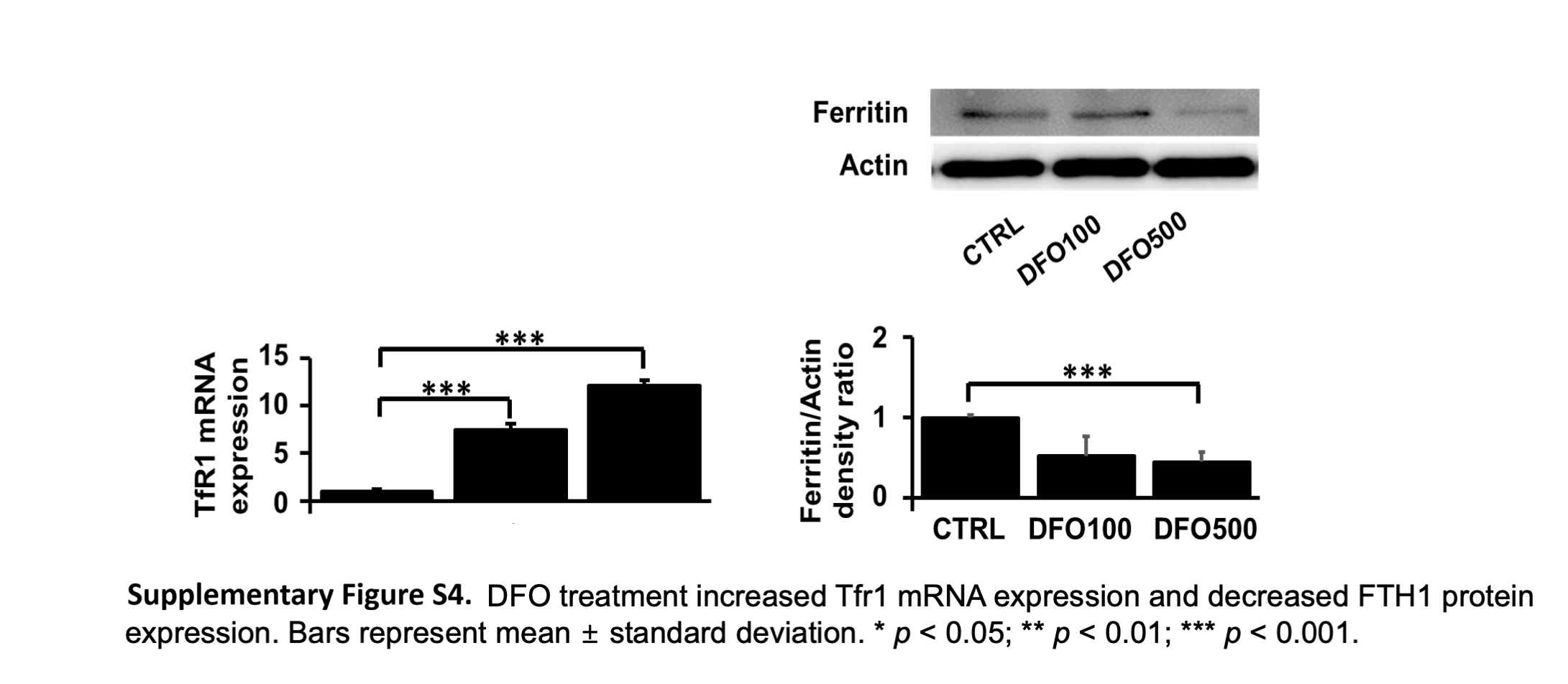

Supplement: Supplementary file 1 [file nutrients-18-02321-s001.zip › Supplementary figure S4.tiff]

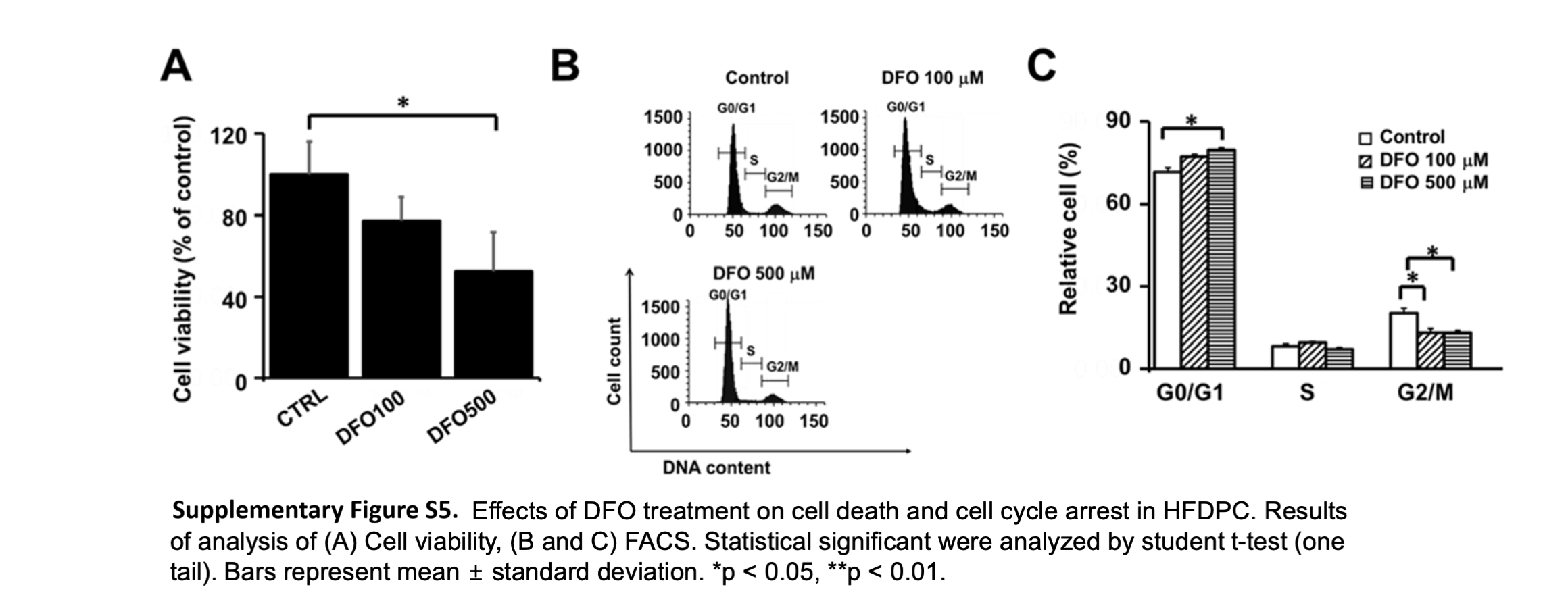

Supplement: Supplementary file 1 [file nutrients-18-02321-s001.zip › Supplementary figure S5.tiff]

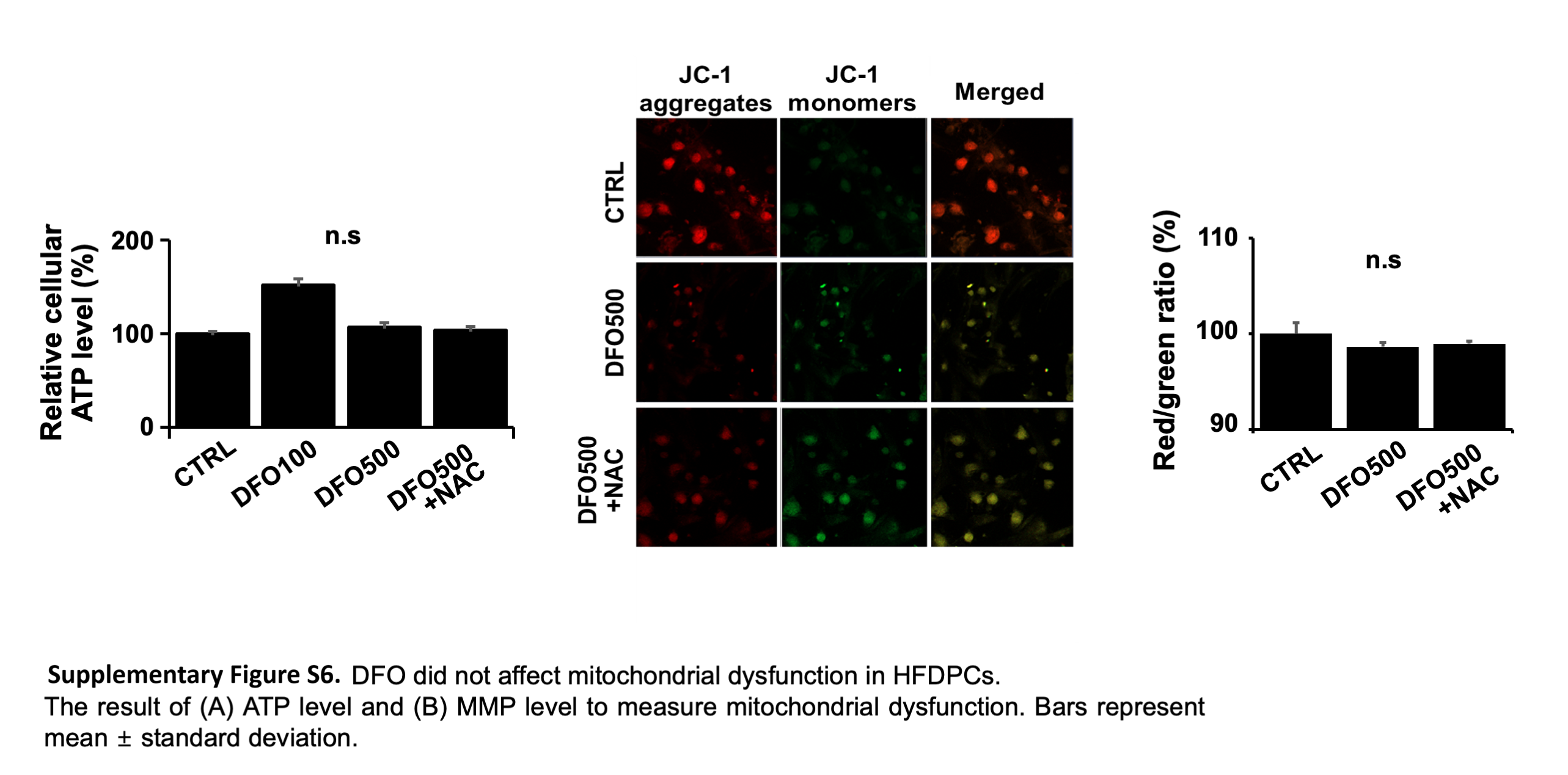

Supplement: Supplementary file 1 [file nutrients-18-02321-s001.zip › Supplementary figure S6.tiff]

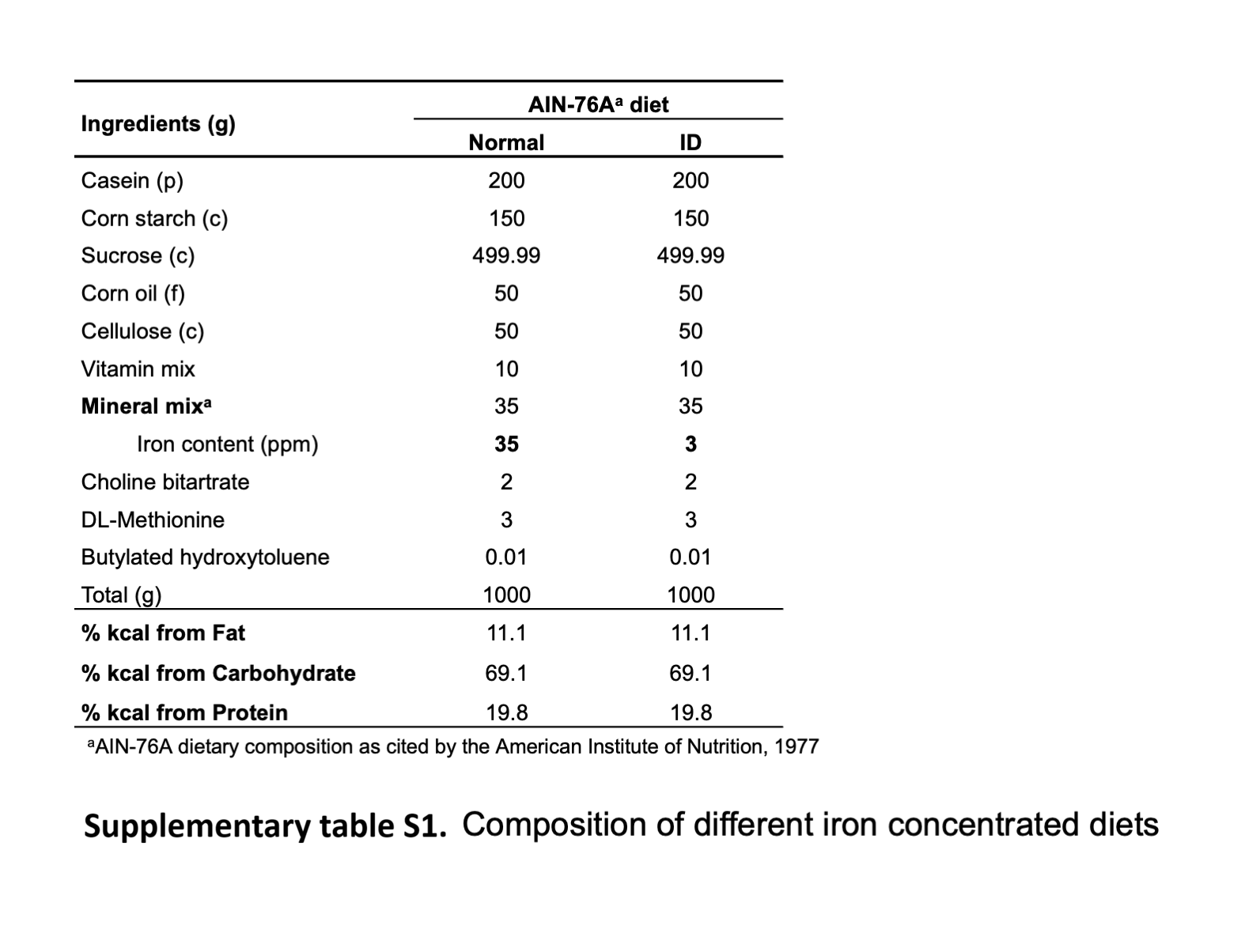

Supplement: Supplementary file 1 [file nutrients-18-02321-s001.zip › Supplementary table S1.tiff]
